# Supplementary material for: Temporal ordering of input modulates connectivity formation in a developmental neuronal network model of the cortex
Source: PLoS One. 2020 Jan 10;15(1):e0226772. doi: 10.1371/journal.pone.0226772 (PMC6953763; doi:10.1371/journal.pone.0226772)
Supplement: S1 Fig — The average rate of input at the start of simulations when the external input exhibits LRTCs (blue) compared with the input randomly shuffled in time (red). This is shown for (A) the first 2% and (B) the first 0.2% of the simulations. Solid lines indicate the mean rate of input across 20 simulations, and the shaded area indicates the standard deviations. (PDF) [file pone.0226772.s001.pdf]

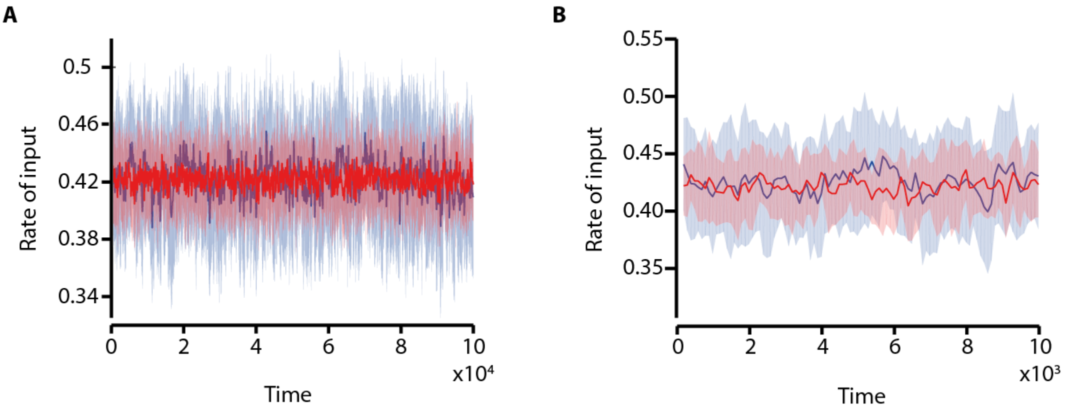

S1 Fig. The initial rate of input to the network is similar whether the external input sequence of IBIs exhibits LRTC or is shuffled. The average rate of input at the start of simulations when the external input exhibits LRTCs (blue) compared with the input randomly shuffled in time (red). This is shown for (A) the first 2% and (B) the first 0.2% of the simulations. Solid lines indicate the mean rate of input across 20 simulations, and the shaded area indicates the standard deviations.
