# Supplementary material for: Temporal ordering of input modulates connectivity formation in a developmental neuronal network model of the cortex
Source: PLoS One. 2020 Jan 10;15(1):e0226772. doi: 10.1371/journal.pone.0226772 (PMC6953763; doi:10.1371/journal.pone.0226772)
Supplement: S2 Fig — (A,B,C) Moving average of the first 1000 IBIs (averaging across 100) for example IBI sequences which exhibit LRTCs (blue, H ≈ 0.7) and are randomly shuffled (red, H ≈ 0.5). (A,B) An example of a IBI sequence which exhibits LRTCs compared with a shuffled sequence. (C) The two IBI sequences which exhibit LRTCs are compared. Note that the full simulations have over 500,000 IBIs so the sequences in these figures equate to approximately the first 0.2% of the simulation. (D,E,F) The proportion of connections in the network across the full simulations for the equivalent IBI sequences shown in the row above. The initial rate of input to the network, reflected in the IBI sequence, does not affect the speed at which the network evolves. (PDF) [file pone.0226772.s002.pdf]

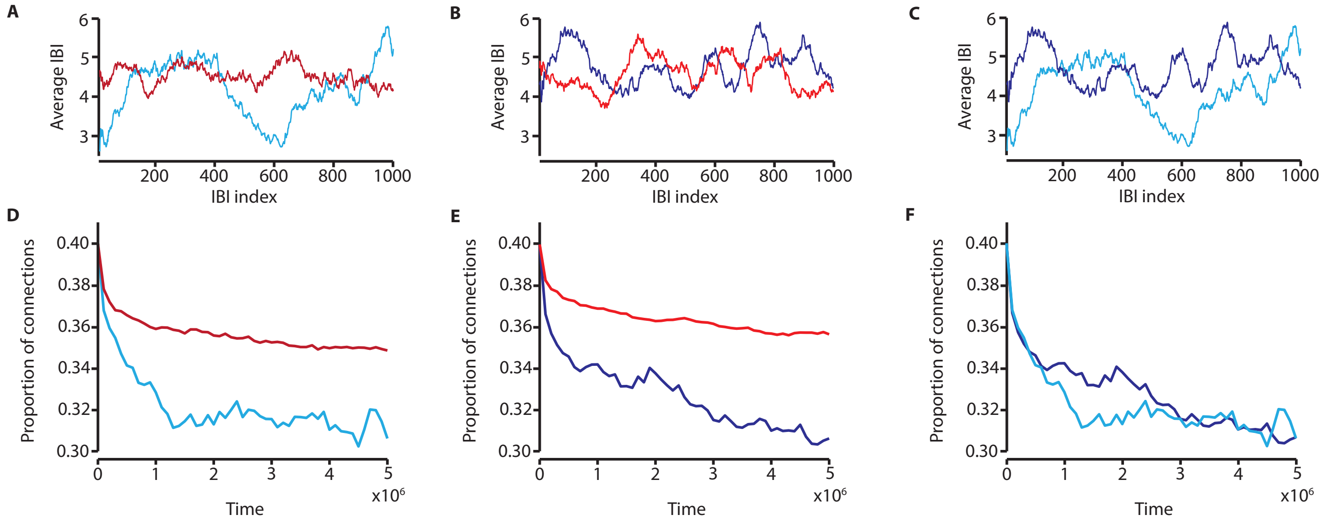

S2 Fig. The speed of emergence of small-world properties at the start of simulations is not related to the average rate of input at the start of the simulations. (A,B,C) Moving average of the first 1000 IBIs (averaging across 100) for example IBI sequences which exhibit LRTCs (blue, $H \approx 0.7$) and are randomly shuffled (red, $H \approx 0.5$). (A,B) An example of a IBI sequence which exhibits LRTCs compared with a shuffled sequence. (C) The two IBI sequences which exhibit LRTCs are compared. Note that the full simulations have over 500,000 IBIs so the sequences in these figures equate to approximately the first 0.2% of the simulation. (D,E,F) The proportion of connections in the network across the full simulations for the equivalent IBI sequences shown in the row above. The initial rate of input to the network, reflected in the IBI sequence, does not affect the speed at which the network evolves.
