# Supplementary material for: Temporal ordering of input modulates connectivity formation in a developmental neuronal network model of the cortex
Source: PLoS One. 2020 Jan 10;15(1):e0226772. doi: 10.1371/journal.pone.0226772 (PMC6953763; doi:10.1371/journal.pone.0226772)
Supplement: S3 Fig — Changes in network parameters with the networks driven by burst input with identical IBI distributions (across all simulations in this figure) but with temporal correlations with different Hurst exponents: H ≈ 0.5 (red), H ≈ 0.6 (purple), H ≈ 0.7 (blue), and H ≈ 0.8 (black). (A) The proportion of connections in the network, (B) the normalised clustering coefficient, (C) the normalised mean path length (note for H ≈ 0.5 and 0.6 the mean path length is equal to one throughout) and (D) the small-world index across the course of the simulations. (E) The average in degree distributions at the end of the simulations. The solid lines indicate the mean across 20 simulations, and the shaded area the standard deviation. Fig 3 demonstrated that the speed of emergence of small-world properties is dependent on the magnitude of the Hurst exponent. However, the IBI distribution in these simulations was not identical as is the case here. This confirms that the differences in the evolution of network parameters is related to the magnitude of the Hurst exponent rather than the IBI distribution. (PDF) [file pone.0226772.s003.pdf]

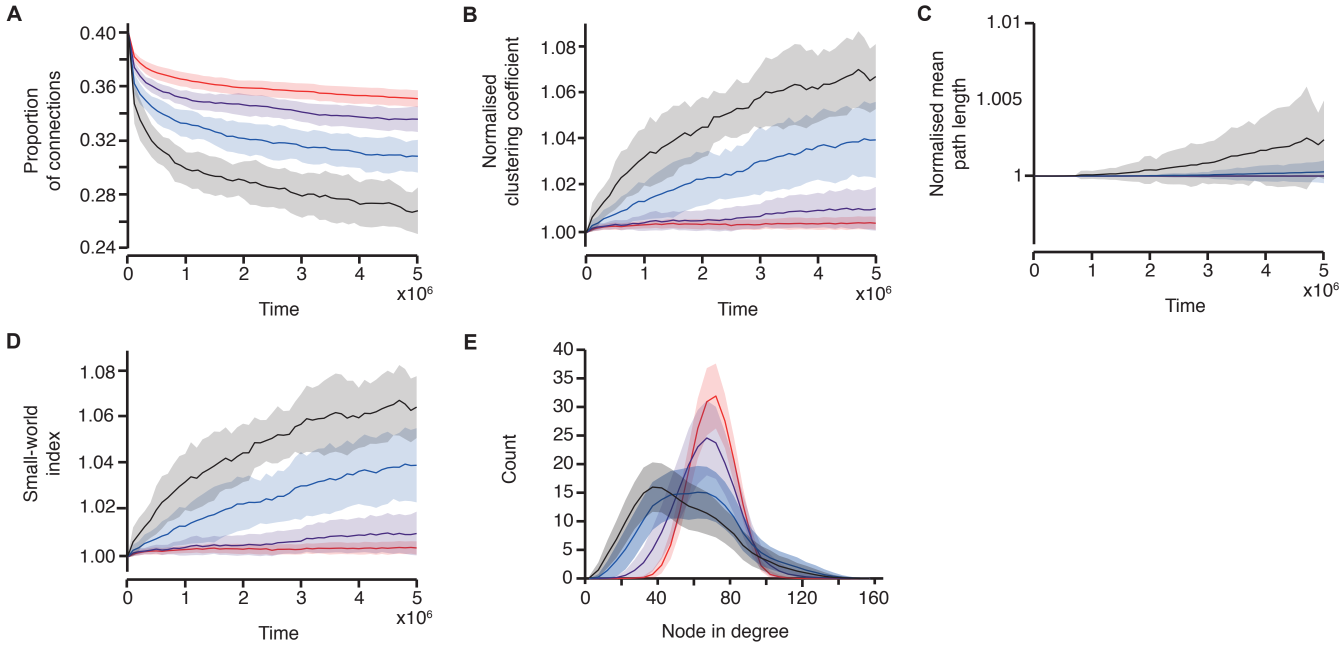

S3 Fig. The rate of network evolution is related to the Hurst exponent of the driving input independent of the overall IBI distribution. Changes in network parameters with the networks driven by burst input with identical IBI distributions (across all simulations in this figure) but with temporal correlations with different Hurst exponents: $H \approx 0.5$ (red), $H \approx 0.6$ (purple), $H \approx 0.7$ (blue), and $H \approx 0.8$ (black). (A) The proportion of connections in the network, (B) the normalised clustering coefficient, (C) the normalised mean path length (note for $H \approx 0.5$ and 0.6 the mean path length is equal to one throughout) and (D) the small-world index across the course of the simulations. (E) The average in degree distributions at the end of the simulations. The solid lines indicate the mean across 20 simulations, and the shaded area the standard deviation. Fig. 3 demonstrated that the speed of emergence of small-world properties is dependent on the magnitude of the Hurst exponent. However, the IBI distribution in these simulations was not identical as is the case here. This confirms that the differences in the evolution of network parameters is related to the magnitude of the Hurst exponent rather than the IBI distribution.
