# Supplementary material for: Temporal ordering of input modulates connectivity formation in a developmental neuronal network model of the cortex
Source: PLoS One. 2020 Jan 10;15(1):e0226772. doi: 10.1371/journal.pone.0226772 (PMC6953763; doi:10.1371/journal.pone.0226772)
Supplement: S4 Fig — Changes in the network parameters with different values of the decay constant of spike timing, τ. τ was varied between (A,B,C,D) τ = 5 and (E,F,G,H) τ = 15. For lower values the network changes are small but small-world properties emerge, and the rate of this emergence is dependent on the Hurst exponent. For higher values of τ with H ≈ 0.7, 0.8 the network becomes disconnected leading to a break down of the small-world properties. (A, E) The proportion of connections in the network, (B, F) the normalised clustering coefficient, (C, G) the small-world index, and (D, H) the number of components across the course of simulations with H ≈ 0.5 (red), H ≈ 0.6 (purple), H ≈ 0.7 (blue), and H ≈ 0.8 (black). Note that in (D) the number of components is equal to one throughout all simulations. Solid lines indicate the mean across 20 simulations, and the shaded area the standard deviation. (PDF) [file pone.0226772.s004.pdf]

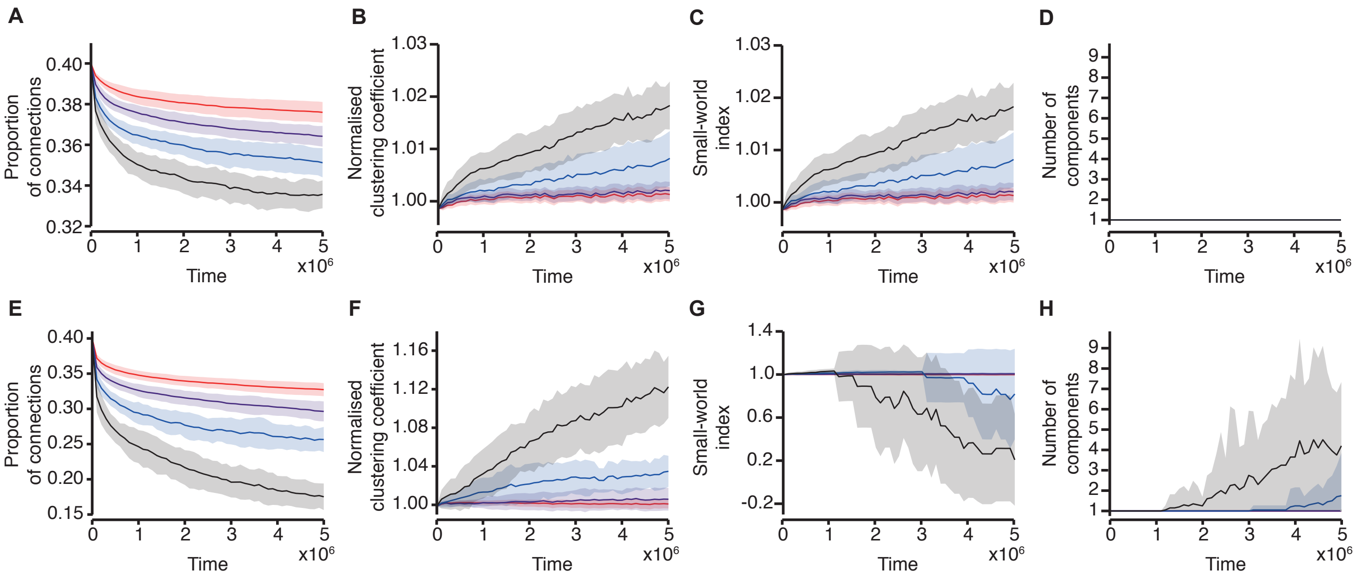

S4 Fig. Network evolution varies with the decay constant of spike timing. Changes in the network parameters with different values of the decay constant of spike timing, τ. τ was varied between (A,B,C,D) $\tau = 5$ and (E,F,G,H) $\tau = 15$. For lower values the network changes are small but small-world properties emerge, and the rate of this emergence is dependent on the Hurst exponent. For higher values of τ with $H \approx 0.7, 0.8$ the network becomes disconnected leading to a break down of the small-world properties. (A, E) The proportion of connections in the network, (B, F) the normalised clustering coefficient, (C, G) the small-world index, and (D, H) the number of components across the course of simulations with $H \approx 0.5$ (red), $H \approx 0.6$ (purple), $H \approx 0.7$ (blue), and $H \approx 0.8$ (black). Note that in (D) the number of components is equal to one throughout all simulations. Solid lines indicate the mean across 20 simulations, and the shaded area the standard deviation.
