# Supplementary material for: Temporal ordering of input modulates connectivity formation in a developmental neuronal network model of the cortex
Source: PLoS One. 2020 Jan 10;15(1):e0226772. doi: 10.1371/journal.pone.0226772 (PMC6953763; doi:10.1371/journal.pone.0226772)
Supplement: S5 Fig — Changes in the network parameters with different values of the decay constant τL. τL was varied between (A,B,C,D) τL = 50 and (E,F,G,H) τL = 150. For low values of τL the network changes are very small but small-world properties start to emerge with H ≈ 0.8. For higher values of τ with H ≈ 0.7, 0.8 the network starts to become disconnected during the course of the simulations. However, there is still a clear distinction in the changes in the proportion of connections and normalised clustering coefficient with different values of the Hurst exponent. (A, E) The proportion of connections in the network, (B, F) the normalised clustering coefficient, (C, G) the small-world index, and (D, H) the number of components across the course of simulations with H ≈ 0.5 (red), H ≈ 0.6 (purple), H ≈ 0.7 (blue), and H ≈ 0.8 (black). Note that in (D) the number of components is equal to one throughout all simulations. Solid lines indicate the mean across 20 simulations, and the shaded area the standard deviation. (PDF) [file pone.0226772.s005.pdf]

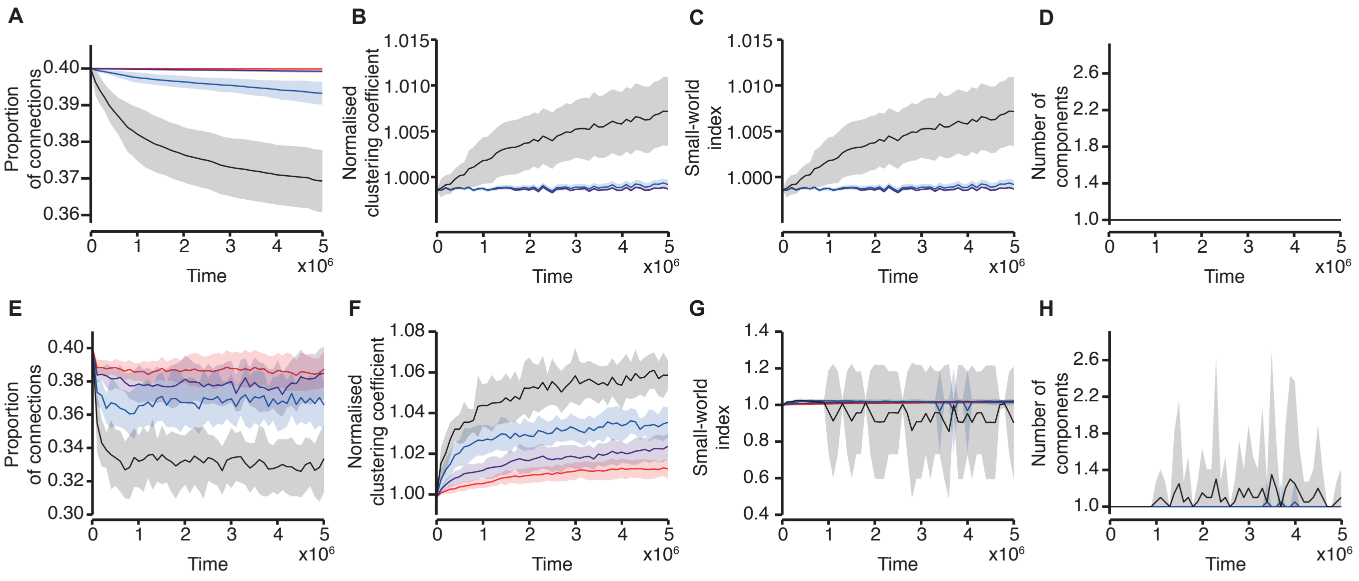

S5 Fig. Network evolution varies with the decay constant of the likelihood of gaining or losing connections. Changes in the network parameters with different values of the decay constant τ_L. τ_L was varied between (A,B,C,D) $\tau_L = 50$ and (E,F,G,H) $\tau_L = 150$. For low values of τ_L the network changes are very small but small-world properties start to emerge with $H \approx 0.8$. For higher values of τ with $H \approx 0.7, 0.8$ the network starts to become disconnected during the course of the simulations. However, there is still a clear distinction in the changes in the proportion of connections and normalised clustering coefficient with different values of the Hurst exponent. (A, E) The proportion of connections in the network, (B, F) the normalised clustering coefficient, (C, G) the small-world index, and (D, H) the number of components across the course of simulations with $H \approx 0.5$ (red), $H \approx 0.6$ (purple), $H \approx 0.7$ (blue), and $H \approx 0.8$ (black). Note that in (D) the number of components is equal to one throughout all simulations. Solid lines indicate the mean across 20 simulations, and the shaded area the standard deviation.
