# Supplementary material for: Temporal ordering of input modulates connectivity formation in a developmental neuronal network model of the cortex
Source: PLoS One. 2020 Jan 10;15(1):e0226772. doi: 10.1371/journal.pone.0226772 (PMC6953763; doi:10.1371/journal.pone.0226772)
Supplement: S6 Fig — (A,E,I,M) The proportion of connections in the network, (B,F,J,N) normalised clustering coefficient, (C,G,K,O) normalised mean path length and (D,H,L,P) small-world index across the course of 20 simulations with a network size of (A-D) N = 100, (E-H) N = 500, (I-L) N = 1000, and (M-P) N = 2000. The networks are driven with burst dynamics which exhibit LRTCs (H ≈ 0.7, blue), compared with the same input randomly shuffled in time (red). Solid lines indicate the mean across the 20 simulations and the shaded area indicates the standard deviation. Results are shown on the same scale for comparison. (PDF) [file pone.0226772.s006.pdf]

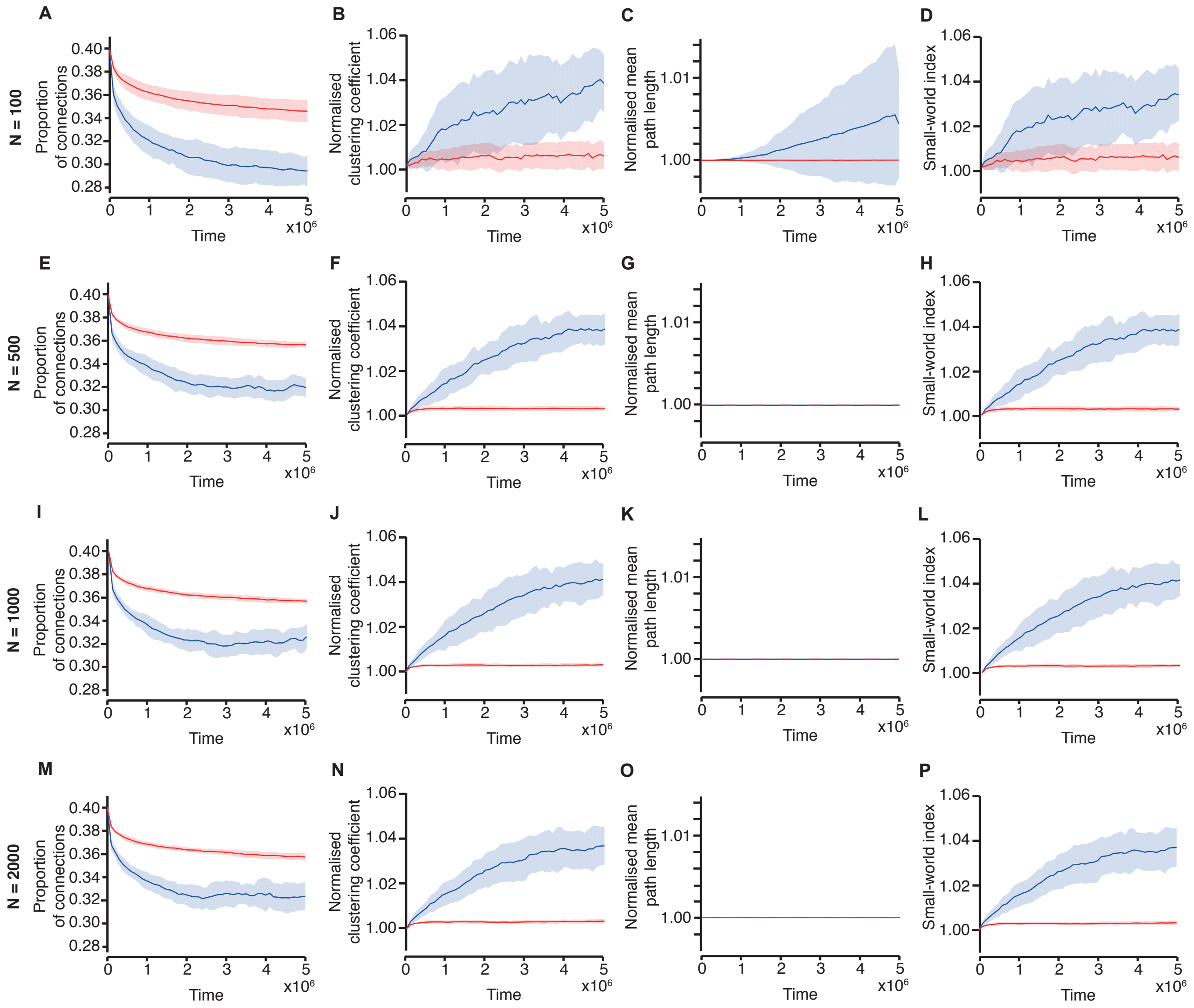

S6 Fig. Changes in connectivity in relation to the size of the network. (A,E,I,M) The proportion of connections in the network, (B,F,J,N) normalised clustering coefficient, (C,G,K,O) normalised mean path length and (D,H,L,P) small-world index across the course of 20 simulations with a network size of (A-D) N=100, (E-H) N=500, (I-L) N=1000, and (M-P) N=2000. The networks are driven with burst dynamics which exhibit LRTCs ($H \approx 0.7$, blue), compared with the same input randomly shuffled in time (red). Solid lines indicate the mean across the 20 simulations and the shaded area indicates the standard deviation. Results are shown on the same scale for comparison.
