# Supplementary material for: Temporal ordering of input modulates connectivity formation in a developmental neuronal network model of the cortex
Source: PLoS One. 2020 Jan 10;15(1):e0226772. doi: 10.1371/journal.pone.0226772 (PMC6953763; doi:10.1371/journal.pone.0226772)
Supplement: Attachment — Submitted filename: Hartley_response_to_ploscompbio_reviews.pdf [file pone.0226772.s007.pdf]

Dear Editor,

Re: Temporal ordering of input modulates connectivity formation in a developmental neuronal network model of the cortex

Thank you for your request for clarification on how we have changed our manuscript following the reviewer's comments from our submission to *PLOS Computational Biology*.

We substantially revised the manuscript in light of the reviewers' comments before we submitted our work to *PLOS One*. We have copied the reviewers' comments below and given a response to each in the blue text. In changing the manuscript text and adding extra sections to the results to address these concerns, we believe we have substantially improved the manuscript.

Please let me know if you would like any further clarification.

With best wishes,

Dr Caroline Hartley

Reviewer #1: This is a simulation-based study of the emergence of connectivity patterns in a network of excitatory leaky integrate-and-fire neurons with spike timing dependent plasticity. The network is driven by bursty inputs and statistics of connectivity recorded. The authors describe the emergence of small-world-network-like topology with hub neurons. While the interplay of activity and plasticity is interesting in general, I did not find the results of this study convincing. The authors do not present an analysis of how robust their results are to changes in model parameters and assumptions. An analytical understanding of the simulation results is lacking. Without these, it is hard to say what concrete predictions the model makes.

These concerns were reiterated by the reviewer below and so we respond to them below.

Major comments:
1) The results are presented using a variety of measures, such as clustering coefficient and small-world index. However, the changes are relatively small in magnitude (e.g. a clustering coefficient going from 1.00 to 1.05). It is unclear how we should interpret these small effects.

We have added a note to the discussion about the magnitude of the effect size in terms of the absolute value being relatively small (line 308). However, we note that given the low standard deviation across the simulations, this provides evidence of a clear difference between the results when the model is driven by the different types of input.

2) There is not a thorough investigation of the results or a simplifying intuition provided. Basic questions include: how important are recurrent interactions versus external driving input? What happens if neurons are not all driven by the same input? What other input statistics would lead to similar results? Attempts to address questions like this would help provide more general intuitions to guide future studies.

Due to these concerns raised by this reviewer and the second reviewer we conducted a number of extra simulations with different parameters and different input to the model. We investigated varying the network size and density, adding a new section to the results (lines 209-225) and Figures 5 and S6. We investigated the effect of periodic input (Figure 2F). We added further discussion to the limitations sections of the results. Please see also our responses to reviewer 2 who asked for more specific investigations.

3) There are some questionable modeling assumptions, like providing identical external input to every neuron and instantaneously adjusting synaptic weights based on the proportion of connections in the network (a non-local synaptic weight update). Neither of these seems well-motivated.

We added further explanation as to why the synaptic weights are adjusted based on the proportion of connections (lines 61-66). This ensured neuronal firing did not die out or saturate the network and is akin to homeostatic plasticity, a biological observed phenomenon.

Reviewer 2

Scale of the model vs. scale of the evidence

I see a fundamental problem here: the empirical evidence (that serves as motivation according to the authors) comes from EEG data. But the model presented consists of spiking integrate and fire neurons. I find this to be a mismatch on the scale of analysis (EEG signal being a function of millions of individual neurons activity).

As a way out, I see two alternative options, although they might actually imply large restructuring of the work: either rephrasing the motivation (avoiding such a strong justification from EEG and showing evidence at the cellular scale), or using a model that represents the EEG signal more closely than spiking neurons (which). Even more so given that the context given by the authors (author summary) is focused on EEG data obtained in humans. The extrapolation from EEG recordings in humans to integrate and fire neuron models is fairly speculative. For example, in line 131-133 (‘’varying Hurst exponent..”) the authors compare their results to infants data: this comparison incurs in the same inconsistency pointed above (the scale of the data -EEG- is not comparable to the one in the model - spiking neurons). The manuscript does not discuss this mismatch at all.

To lines 53-60 we added the following text:

> Whilst this work is motivated by the observation of LRTCs in the EEG of preterm infants, i.e. an observation at the macroscopic scale, LRTCs have been demonstrated at multiple levels of the nervous system in adults, including in the spontaneous spiking of individual neurones. Although the external input which provides the LRTCs within our model is on the macroscopic scale, with bursts originating from non-cortical sources such as the subplate, our focus here is on the effect such macroscopic phenomenon has on connectivity formation at the microscopic (neuronal) scale.

We also added further comments about this to the discussion (lines 272-276).

Methodological aspects

- Why did the authors choose a spiking neuron model to represent neural activity? Specially taking into account that the empirical evidence motivating the work comes from EEG recordings.
  See previous comment above.
- As a null model to test the effect of the temporal order of input activity, a shuffled sequence is passed to the network.

- – Why is this a good null model?
  This preserves the inter-burst interval distribution whilst changing the temporal correlations in ordering of the inter-burst intervals. This has been clarified in the introduction line 45 and the results line 137.
- – Have you tried other dynamical regimes less extreme than random shuffling? It would be useful to, at least, discard the possibility that other simple types of structure lead to same effect (e.g., periodic oscillations.). The effect might be also achievable with other structure of the input and I think it would be specially meaningful to explore this considering that all the effect sizes shown are rather small.
  We have now investigated the effect of periodic input (lines 136 – 146, Figure 2F), demonstrating similar results to the case with shuffled input.

- Initial connectivity is 40%:
  - – Why? Isn't it too high? Specially if the individual units of the network are supposed to be single neurons.
  - – Is there any estimation for human connectivity density at that stage?
    We have now investigated variations in network initial connection density (lines 209 – 225, Figure 5).

- All neurons receive the same input:
  - – Why did you make this choice?
  - – Does that resemble any empirical evidence?
  - – Have you tried at least some coarse exploration of this parameter (such as providing with input to 25% or 50% of neurons)?
    We chose to make this assumption as it allowed the dynamics of the external input to be transferred to the system as a whole. This assumption would not be valid to model the entire brain, but we are modelling a very small population of neurones (N=200) and so we chose to do this in order to simplify the model. This assumption and the limitations of making this assumption have been clarified in the discussion (lines 315-320).

- How do you deal with disconnected networks? Do you have to adjust any aspect of your simulation or is your set up just robust to that?
  We did not adjust for this and sometimes the networks did become disconnected. We note this as a limitation (lines 326 – 331).

- The model presented somehow "hardcodes" the weights to be exactly uniformly distributed all the time. How is that biologically motivated?
  I think this should be stated already in the results section (not only in the Methods section, lines 319-322), since it is an important modeling decision worth communicating in a clearer way, in order to make the reader aware of it as soon as possible.
  This was biologically motivated by the phenomenon of homeostatic plasticity. We have now included this in the results section as suggested (lines 61-65) as well as in the Methods section as previously described.

- How do you define the coupling between neurons? It is not specified in the equations.
  Coupling is described in equation (1) in the methods and on lines 379-389.

  On the results
  • In general, effect sizes on network metrics are rather small. What can you tell about that?

We have commented on this in the Results (lines 96-99) and in the discussion (line 308).

• Figure 2:

- – Evolution of path length should be shown, in order to see its contribution compared to the one of the rise in clustering.
  This has now been included (Figure 2C).

- – I find the curves corresponding to the two conditions (with/without LRTCs) not to be completely comparable in terms of network metrics (since they differ in network density). Please take a look at [1] for a more detailed description of the problem. In other words, can you completely discard the possibility that the observed effect (rise in small-worldness driven apparently by rise in clustering) is due to the drop in density?
  We compared the network metrics for each condition (with/without LRTCs) with random networks (50 per condition per time point – see methods) of the same network density. The metrics are therefore normalised to take into account this difference in density.

I understand that the network metrics are normalized with respect to a random graph of same size and density, but that does not exclude by itself the fact that for different densities the expectation of certain metrics might be different and non trivial biases appear when comparing networks of different size/density (see [1]). Concretely, for example, it would be informative to, in addition to the two conditions of interest, include (as a null/control condition) a curve where the network metrics are computed but for a null network that matches the density of the network evolving under the LRTCs condition.
We agree that it is important to compute these networks for a 'null network'. This is the function of the random graph of the same size and density.

- – The degree distributions do not clarify whether they are of in or out neighbors (network is directed). In the methods section (line 414) says that in/out degrees are considered separately, but it is not shown in the figures. I find it is worth making that point clear (either in the caption or in the plot itself).
  This has been clarified in the Figure captions, and the out degree distribution has been additionally added to Figure 3F.

- – Long term behavior of the model:
  ∗ How does the model behave for longer simulations? Does it loose all the connections? ∗ How did you define the simulation length?
  The simulations take a considerable amount of computational power (taking several days to run each). We ran the simulations until the proportion of connections appeared to somewhat plateau. We have not tried to run longer simulations due to the computational power needed.

- For illustrative purposes, it would be very informative to actually display example graphs resulting from the model.
  Given the number of connections and nodes we do not feel that these plots are very informative, and they can only show a snapshot of network connectivity (an example plot is below). The network metrics for the average graphs are able to give a much better picture of network evolution. We therefore chose not to include them.

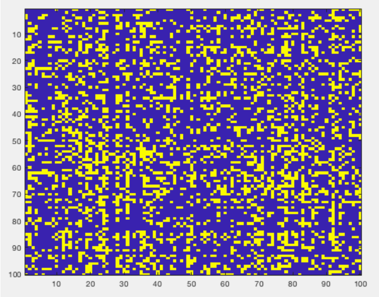

- Beyond clustering, path length and small-worldness, have you looked at other network proper- ties (such as modules) also typical of brain networks?
  We have not looked at the properties as we chose to concentrate on the small-worldness and the presence of hub neurones, two properties which have been shown to evolve early in development.

- How robust are the results with respect to network size and density?
  We have added a new section to the results looking at this (lines 209-225), with the addition of Figure 5 and Figure S6.

Interpretations and Discussion

- Is there any mechanistic explanation of why this dynamical input (bursty activity with LRTCs) might induce such a network topology?
  As we have discussed, the presence of LRTCs seems to change the rate of emergence of the small-world topology rather than inducing it uniquely. This is likely due to the temporal dynamics of the input, for example, with short inter-burst intervals likely to co-occur. However, this is speculative.

- Do the authors have any clue about how/why bursts are generated?
  We have included references to previous biological and computational papers describing this (lines 265, 270-272, 282).

- Two modeling decisions/assumptions that I think should be explicitly addressed (at least in the ”Limitations” section of the discussion):
  – No geometrical constrain/information is taken into account by the model.
  – Connections can be formed between arbitrary units independently of their distance (be topological or geometrical).
- I find the statement in lines 278-280 a bit too bold for being a speculation. The extrapolation is rather large and after all, in my opinion, not really necessary. For instance, in order to claim something like that, there should be evidence of connectivity patterns at that precise scale showing small world properties.
  This referred to the last line of the summary where we had previously written “LRTCs play an essential role in this connectivity formation”. We have changed this to “LRTCs may play an important role in this connectivity formation warranting further investigation”.

Minor phrasing aspects

- Abstract: ”small world connectivity and hub neurons emerged” → clarify that these are struc- tural (and not functional) properties in this case.
  This was clarified.

- Line 48: authors claim that the emergence of small-world connectivity ”will facilitate infor- mation processing”. Yet, they don’t test such a hypothesis. I understand that the authors

state this as a plausible implication based on the cited literature. Still, the results presented show only topological outcomes. I suggest either removing such statement (i.e., simply consider the topological consequences without making any inference about information processing) or actually testing information processing in this specific context. The reason for that is that, first, efficiency is not clearly defined by the authors; second, and most importantly, it could be the case that small-worldness is a necessary condition for efficient information processing, but not a sufficient one. So, the network resulting from the presented model might potentially, at the same time, have small-world properties and not be efficient.
This statement was removed (it was the last sentence of the introduction, now line 49).

- Line 405: ”... neural networks have similar mean path length to a random network (of the same size)...” → AND DENSITY.
  This was amended (sentence now on line 470).
